# The parallel lives of alpha_1_-antitrypsin deficiency and pulmonary alveolar proteinosis

**DOI:** 10.1186/1750-1172-8-153

**Published:** 2013-09-30

**Authors:** Bruce C Trapnell, Maurizio Luisetti

**Affiliations:** 1 Cincinnati Children Hospital Medical Center, Cincinnati OH, USA; 2 Department of Molecular Medicine, Pneumology Unit, San Matteo Hospital Foundation, University of Pavia, Piazza Golgi 1, Pavia 27100, Italy

**Keywords:** Alpha1-proteinase inhibitor, Pulmonary emphysema, Whole lung lavage

## Abstract

In 1963, five cases of alpha1-antitrypsin deficiency were reported in the scientific literature, as well as an attempt to treat pulmonary alveolar proteinosis by a massive washing of the lung (whole lung lavage). Now, fifty years later, it seems the ideal moment not only to commemorate these publications, but also to point out the influence both papers had in the following decades and how knowledge on these two fascinating rare respiratory disorders progressed over the years. This paper is therefore not aimed at being a comprehensive review for both disorders, but rather at comparing the evolution of alpha1-antitrypsin, a rare disorder, with that of pulmonary alveolar proteinosis, an ultra-rare disease. We wanted to emphasize how all stakeholders might contribute to the dissemination of the awareness of rare diseases, that need to be chaperoned from the ghetto of neglected disorders to the dignity of recognizable and treatable disorders.

## Introduction

**Figure 1:**
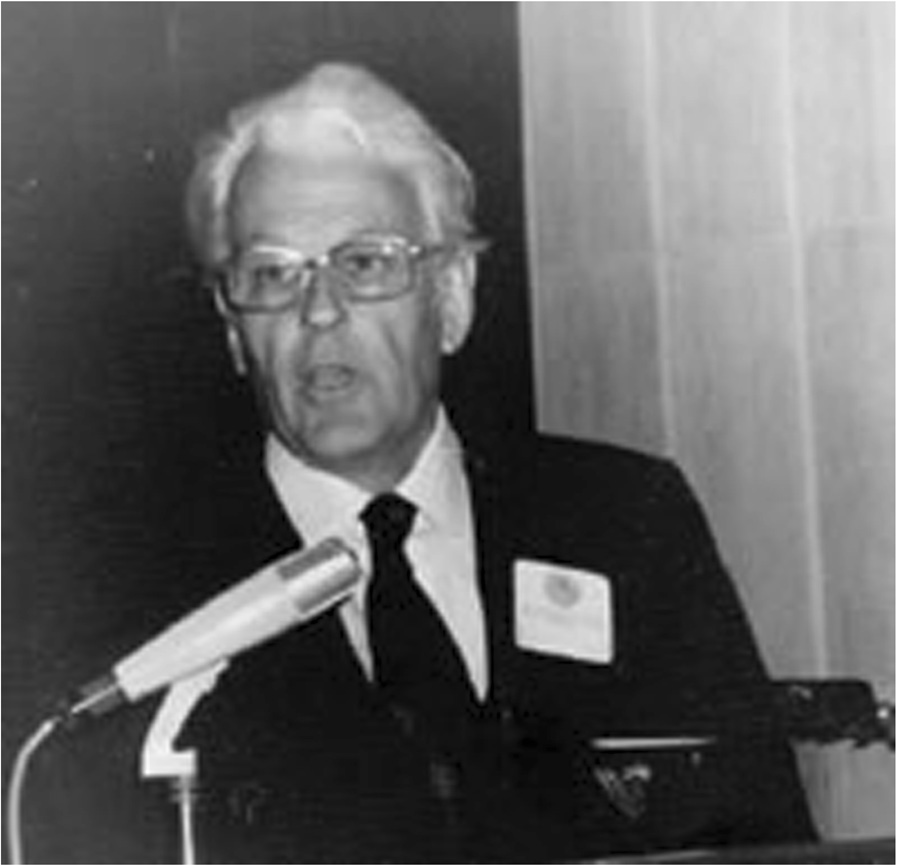
Carl-Bertil Laurell (Courtesy of eALTA Grifols).

**Figure 2:**
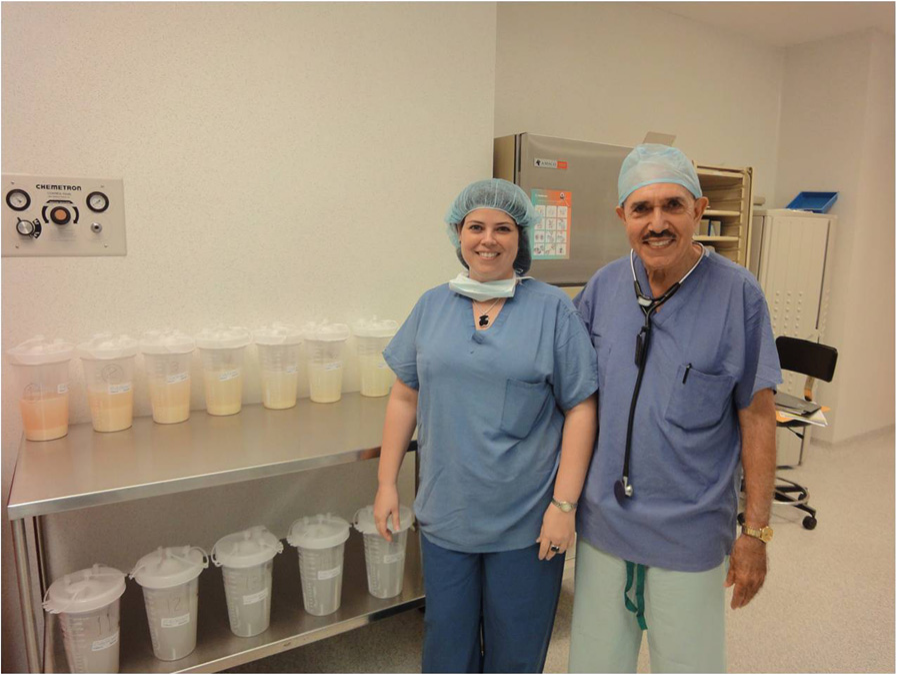
José Ramirez Rivera with a young pulmonary staff member at University of Puerto Rico, 2012.

In 1963 the world was in a state of fermentation, which eventually exploded in the second half of the sixties on both sides of the Atlantic Ocean and in different areas: affecting social life, the arts and protest movements. See Additional file 1 for a historic landscape. The world of Science in the meantime was thrilled by the first human organ transplants for liver, lung, and kidney. Two much less thrilling articles, but which had a great impact in the following years, were published in 1963. Carl- Bertil Laurell (Figure 1) and Sten Eriksson at the Malmö University in Sweden reported on five cases of serum alpha1-antitrypsin (AAT) absence in the electrophoresis; interestingly three out the five carriers suffered from pulmonary emphysema [1,2]. Almost simultaneously, three physicians from the Johns Hopkins University, led by Jose Ramirez-Rivera, (Figure 2) experimented with the segmental flooding technique by means of an endobronchial catheter [3] to remove the accumulated material within the airspaces in a young male affected by pulmonary alveolar proteinosis, a mysterious disease which was first described five years earlier by Rosen and colleagues [4]. At the moment of publication, neither paper was enthusiastically acclaimed, but now fifty years after their original release it seems the ideal moment not only celebrate their publication, but most of all point out the influence both papers had in the following decades and how knowledge on these two fascinating rare respiratory disorders progressed over the years.

### From sixties to eighties

The ten years following this discovery were marked with events of a lifetime for alpha1-antitrypsin deficiency (AATD) in Sweden. In his captivating review [5], Robin Carrell told how the original description of Laurell & Eriksson promoted an extraordinary, lively and productive environment in Sweden, and in Malmö in particular. Kjell Ohlsson, Jan Olof Jeppson, Magne Fagerhöl and Diane Cox, the latter arriving in Malmö from Norway and Canada, respectively, focused their work on the explanation of the complex electrophoretic heterogeneity of AAT, eventually contributing to the development of Pi nomenclature for AAT variants [6-8]. In the meantime, Christeer Larson provided evidence for the interaction of smoking with AATD [9], thus contributing to the current oxidation stress/proteinase imbalance hypothesis of the pathogenesis of emphysema, and Tomas Sveger performed the Swedish newborn national screening for AATD, a hallmark event in the epidemiology of the disorder [10]. To complete the Scandinavian perspective, the same investigators detected the inclusion of AAT within hepatocytes of AATD subjects with liver disease [11], a finding that was however anticipated a few years before by Dr Sharp and colleagues in the US [12].

The next decade was equally productive for AATD research. On the one hand, the reactive site of AAT was identified, as well as its vulnerability to oxidant stress [13,14]. On the other hand, the crystallographic structure of AAT was elucidated and the first hypothesis was proposed that AATD was due to a structural perturbation hampering the extracellular secretion of the mature protein [15,16]. From the molecular perspective, the mid-eighties were marked by successful cloning and sequencing of the human AAT gene (currently named SERPINA1), and the identification of the point mutation underlying the AATD Z variant [17,18]. In the meantime, reports of longitudinal studies progressively improved our knowledge on the natural history and clinical phenotypes of individuals with AATD-associated clinical conditions [19]. The decade ended with the hallmark study on the feasibility of purified protein replacement therapy in AATD deficiency subjects [20].

The two decades following the original whole lung lavage (WLL) description were not so eventful. The major advance achieved during this period, as reported by Seymour and Presneill in their review [21], was the progressive improvement of the original washing technique described by Ramirez-Rivera, which matured into the WLL as we know it today [22]: the adoption of general anesthesia, the progressive increase of fluid volume, the usefulness of chest percussion, ending with the successful lavage of both lungs in the same session. Most papers published in this period were anecdotal studies of the disorder, they did however contribute to the expansion of our knowledge. Interestingly, some of these reports, although not directly addressing the pathogenesis of PAP, pointed to some aspects of the disease heterogeneity and development. The induction of proteinosis in the animal model of silica exposure [23], and the report on PAP occurrence in a subjects with heavy exposure to aluminum dust [24], as well as the report on rare cases of PAP in patients with hematological malignant disease [25] foresaw some of the forms of secondary PAP. The report on familial clustering of cases of PAP [26] described the occurrence of hereditary proteinosis, whereas the presence of newborn PAP as a cause of neonatal respiratory distress syndrome [27] first reported the so called PAP-like forms due to surfactant protein genetic abnormalities. Pathogenesis of the most common form of PAP, referred to as idiopathic, was unknown at that time, and bound to remain so for several decades, but David W Golde in 1976 focused his attention on the defective activity of lipid-laden macrophages [28], a cell that eventually was recognized to play a pivotal role in the development of PAP. In their paper published in 1984, William Claypool and colleagues [29] reviewed the current status of knowledge on pathogenesis and management of PAP: they carefully described their experience with 34 PAP patients, the single lung, whole lavage technique, and reviewed possible steps in PAP pathogenesis, concluding that the pathophysiology of surfactant disorders such as PAP would challenge scientists and physicians in the future: this held true for at least 10 more years.

### The next twenty years : form nineties to 2010

Progress in the study of AATD from 1990 to 2010 proceeded in different directions. The AATD disease mechanism underwent progressive clarification. The most frequent AAT variant associated with severe deficiency, Glu^342^Lys, also referred to as PI*Z, was shown to form polymers and accumulate within hepatocytes [30], causing the deficiency in the bloodstream. This led to the hypothesis of a divergent mechanism for lung and liver disease in AATD: a deficiency mechanism (“loss-of-function”) in lung disease, and an add-on, related to the misfolding of the protein (“gain-of-function”), a conformational mechanism in liver disease [31,32]. This Manichean view was however complicated by evidence that PI*Z polymers may also be detected and likely produced within the lung [33], thus suggesting that the add-on mechanism could also contribute to lung disease. A large series of AATD patients were studied in the United States and United Kingdom during this period, and greatly contributed to our knowledge on the clinical presentation and natural history of lung disease associated with AATD, in terms of mortality, FEV_1_ decline, and exacerbations [34-36], as well as the associated liver disease [37]. The epidemiology of AATD received great attention after the publication of the worldwide analysis by Fredrick de Serres: in his estimation, albeit in part refined in numerous subsequent publications, ca. 30,000,000 individuals are at risk for adverse health effects due to different AATD genotypes [38]. Replacement therapy with *i.v.* infusion of purified human plasma protein was licensed in the last decade of the Twentieth Century, and progressively became available. As a result, thousands of patients with lung disease associated with AATD have been safely treated [39,40]: a meta-analysis of observational studies confirmed efficacy with the decreasing decline of lung function in treated patients with an initial FEV_1_ between 30 and 65% predicted [41]. A number of alternative treatments for AATD have been proposed, ranging from inhalation therapy to recombinant and transgenic AAT, from gene therapy to regenerative medicine [42-45]: none of these options has so far gone beyond the experimental stage. AATD played a critical role in the last two decades in building one of the most long-lived and respected hypotheses for the development of common pulmonary emphysema: the theory of an imbalance between proteinases and proteinase inhibitors, took shape, which evolved over the years, with the biochemical evidence of emphysema in subjects lacking AAT [46].

At the beginning of the last 1990’s, compared with AATD, PAP lagged behind in terms of knowledge on pathogenesis. But it quickly made up for lost time: in 1994 two papers demonstrated simultaneously and serendipitously that mice lacking GM-CSF (granulocyte-macrophage colony-stimulating factor) developed a lung disease similar to human PAP [47,48]. These data showed that GM-CSF is critical for surfactant homeostasis in the lung, leading to subsequent studies and evidence that PAP was related to impaired surfactant catabolism by alveolar macrophages [49]. However the etiology of surfactant impairment in PAP remained unexplained until 1999, when Koh Nakata and coworkers demonstrated the presence of polyclonal, neutralizing anti-GM-CSF autoantibodies (GMAbs) in patients with “idiopathic” PAP [50]. Shortly thereafter, the pathogenesis of PAP in GM-CSF-deficient mice was elucidated in a study demonstrating that pulmonary GM-CSF is required for the terminal differentiation of alveolar macrophages [51]. Subsequently, passive transfer studies in non-human primates injected with purified human PAP patient-derived GMAbs provided proof of their role in pathogenesis of PAP in humans (and of the critical role of GM-CSF in terminal differentiation of alveolar macrophages in primates) [52]. These and other studies helped to define the previously designated “idiopathic” PAP as an autoimmune disorder and led to a new classification of surfactant disorders, including secondary PAP and rare forms of hereditary PAP [53]. The progressive evolution and improvements in the WLL technique over the years dramatically changed the natural course of the disease, which was originally charged with a mortality of approximately 30%, it progressively became a disease with a substantially favorable prognosis [21]. In the 70% of PAP patients a single WLL is enough to provide a prolonged period free of disease and/or symptoms [54]. Although WLL is a relatively safe procedure in experienced hands, it is however an invasive procedure, not exempt from severe complications. Therefore based on novel pathogenesis insights, novel therapeutic options have sprung [55]. To restore appropriate GM-CSF signaling, impaired by the presence of GMAbs, supplementation with exogenous recombinant GM-CSF has been proposed, first by subcutaneous injection, and then by inhalation [56,57]; results were substantially better with the latter delivery method. Considering the mechanisms underlying the autoimmune form of PAP, a biological approach seemed reasonable. An open-label trial investigating Rituximab treatment which depletes the CD20 B-cell population provided intriguing, preliminary results [58].

### Achievements in the first fifty years and expectations for the future

**Figure 3:**
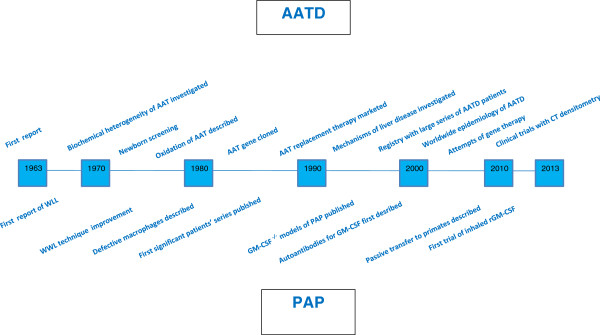
Milestones of the fifty years progress in the knowledge of AATD and PAP.

As with any anniversary, retrospection and introspection are in order. We should address the basic question: what contributions have been made during these 50 years to our understanding and management of these two rare respiratory diseases (Figure 3). The answer is largely positive, although with understandable differences:

•AATD and PAP are both rare respiratory disorders, but with remarkable differences in prevalence, now recognized at: 33/100,000 for AATD and 0.7/1100.000 for PAP [59,60]. PAP thus ranks among ultra-rare diseases (i.e. rare disease with a prevalence < 1/100,000 individuals).

•In spite of this difference, although a formal registry for PAP is not available, published data for more than 1,000 PAP patients are available [61]. On the other hand, two large registries for AATD are active, one in the US (Alpha-1 Research Registry), and the second is an international registry (Alpha One International Registry, AIR) [62], with about a total of 9,500 AATD patients enrolled. Such a large series of patients will contribute to a better understanding of the natural history of both diseases.

•A marked difference is however evident in molecular epidemiology data, since we have a comprehensive view for AATD [63], whereas PAP data are scattered and incomplete.

•Large diagnostic programs for AATD have been established over the last two decades in Western countries [64], with consolidated diagnostic flow-charts for genetic testing, and new programs are currently going to be implemented in Eastern Europe, whereas for PAP we are at the early stage of establishment of reference centers in the US and Europe. However we are at a satisfactory stage, compared with the very recent past.

•Powerful patient-advocacy groups for AATD are active both in the US [65] and Europe, whereas a PAP patient organization is present only in the US [66] and its activity is very limited.

•Thousands of AATD patients are currently on replacement therapy in both the Americas and in Europe; in contrast, WLL is not a standardized procedure, and is available only in selected centers. A worldwide census of centers with experience performing WLL [67] hopefully will represent the first step toward standardizing the procedure.

•Last, but not least: the search for surrogate markers to prove efficacy of replacement therapy in AATD, has greatly contributed to the development of computed tomography-based lung densitometry [68-70], a technique likely to be implemented in common emphysema [71] for testing new potentially active drugs.

Major review articles are available for both disorders: at least three for AATD, covering different aspects of the disease [72-75], and one for PAP (75).

On a final note, we would like to express our expectations for the coming years. The AATD community is anxiously waiting for unbiased proof of efficacy for replacement therapy and, in turn, an alignment in accessibility to therapy among European countries. Research will hopefully address alternatives to plasma purification of AAT, in order to improve efficacy, reduce costs, and broaden availability: inhalation delivery, recombinant AAT, as well as regenerative medicine, and drugs able to correct misfolded AAT are all under active investigation, as stated above. On the other hand, it is desirable that detection programs reduce the huge gap between diagnosed and estimated individuals with severe AATD, making epidemiology data more robust. The path for PAP is understandably longer, but hopefully not winding. Registries, standards of care, networks/centers of excellence, precise epidemiology (does ultra-rare status stem from ignorance?), patient advocacy for PAP are still in the embryonic stage. Lessons from AATD should be extended to PAP, with the hope that it will share the same interest as AATD: biological treatments will hopefully help achieve this goal. This would definitely bring PAP out of the ghetto of neglected diseases, bringing it the parallel with AATD, converging into the dignity of rare diseases with equal awareness. It is hoped that such a process does not require fifty more years.

## Competing interests

The authors declare that they have no competing interests.

## Authors’ contributions

The paper has conceived and written by ML and BCT. They both approved this version to be published.

## Supplementary Material

Additional file 1 Historic landscape. Click here for file
