# Supplementary material for: The parallel lives of alpha1-antitrypsin deficiency and pulmonary alveolar proteinosis
Source: Orphanet J Rare Dis. 2013 Sep 30;8:153. doi: 10.1186/1750-1172-8-153 (PMC3849781; doi:10.1186/1750-1172-8-153)
Supplement: Additional file 1 — Historic landscape. [file 1750-1172-8-153-S1.docx]

THE PARALLEL LIVES OF ALPHA_1_-ANTITRYPSIN DEFICIENCY AND

PULMONARY ALVEOLAR PROTEINOSIS

Bruce C Trapnell ^1^, Maurizio Luisetti ^2^

*Historic landscape*

So young and so busy with games, sports, and their elementary education, not to mention the first of many examinations they would face in their lives, the authors of this essay did not realize at that time that 1963 was a critical year marking the passage of the twentieth century towards a progressive definition of the world as we currently know it. Marshall McLuhan would theorize the following year how technology was about to change and influence the perception of society. The news of the assassination of President John F. Kennedy in Dallas TX, broadcasted on November 22, is the one event we are used to say that everybody remembers exactly where they were and what they were doing the moment when the news came. Gathered around the TV screen with their families in Federalsburg, a small town on the eastern shore of Maryland, USA and in Cantù, a small town in the suburb of Como, Italy, respectively, we were both living not only a tragedy that, after many decades, is still an unhealed wound in the conscience of America and worldwide, but also perhaps the dress rehearsal for the real-time global village, with the planet simultaneously tuned in to the same wavelength.

Many other events happened in 1963 which eventually would have a profound impact on the world community and political life in the coming decades. On June 3 in Rome, Pope John XXIII died, who the previous year had declared the Vatican II Council open, less than ninety years after the Vatican I Council. Rather than discussing theoretical dogmata, the Council addressed the need for an understanding of the signs of the times, as Pope John XXIII emphasized in his opening speech his desire to strengthen the universal ecumenism of the Catholic Church. Ninety sixty three in the United States was rich with events related to the civil rights movement: the JF Kennedy Civil Rights Address, as well as Martin Luther King’s *I have a* *Dream* Speech at the Lincoln Memorial, the assassination of Megdar Evers and the graduation of James Meredith are memorable events which paved the long march: after another 46 years Mr Barack Obama was elected President of the United States, the first member from the African American community. November 1963, with the death of JFK and the South Vietnam coup, resulted in the execution of President Ngo Dinh Diem, marked the escalation of the Vietnam war. The Beatles released their first album *Please Please Me*, and Bob Dylan his second album *The Freewheelin’ Bob Dylan,* both bound to have a profound influence on folk/popular music in the second half of the last century.
